# Spinal Hemiplegia—II

**Published:** 1908-03-14

**Authors:** 


### Medicine.

### SPINAL HEMIPLEGIA—II.

### WITH ANÆSTHESIA ON THE SAME SIDE.

The following is yet a third case of hemiplegia due to compression of one side of the cord; and here again, as in the former two cases, the anæsthesia was upon the *same* side as was the paralysis or paresis.

The patient is a man, aged 25, who used to work in an arsenal. With the exception of an attack of malaria four years ago, he has enjoyed good health. Ten months ago a plank fell upon his left side, striking him in the interval between the last rib and the crest of the ilium. The patient suffered severe pain in the lumbar region immediately after the accident, and although he was able to return to his work a few weeks after the accident, the pain returned, and gradually increased, and was accompanied by partial loss of power in the left arm and left leg, with impairment of sensation in these limbs and no alteration in sensibility in those of the right side.

The man entered a hospital, where he was kept in bed, and treated with mercury and counter-irritation to the spine. In a few weeks he recovered the power of sensation in his arm and leg, lost the pain in his back, and regained strength in the paretic limbs, though neither the arm nor the leg became as strong as it had formerly been. There had been no paresis of the face; no cerebral symptoms; the man denied syphilis; and there was no obvious affection of lungs, heart, kidneys, nor abdominal organs. There is no note as to whether the thoracic and abdominal muscles moved as well on the left side of the body as on the right—a point to which particular attention should be directed in cases of hemiplegia thought to be spinal.

The man returned to work again, but in nine days he was obliged to leave off in consequence of recurrence of pain in the vertebral column. He returned to bed, and for a time improved a little under iron and quinine and cod-liver oil. The improvement was not permanent, and on November 14 he was readmitted to hospital. The pain had now assumed a new character—that of remarkable periodicity. It began at night and lasted for three or four hours with great severity, then subsiding gradually and allowing the man to get to sleep. On awaking in the morning the patient would feel a soreness in the region where the pain had existed the previous night; it seemed to relieve him to sit up, though he was only allowed to move with great caution. Any sudden contraction of the muscles of the left side caused severe pain, similar to that experienced by a man with lumbago who tries to move.

A careful examination of the spine discovered nothing wrong there, but a decided pain was produced by pressure over the left quadratus lumborum muscle, and the act of coughing or sneezing was excruciating. From the fact that the pain had its seat where the injury occurred, and also because it was excited by muscular exertion when the lumbar muscles of the left side were in action, it seemed likely that a great part of the pain might be due to some condition of the muscles themselves, such as rupture of some of the fibres as the result of the injury inflicted by the fall of the plank. There was even at one time a suspicion that the whole thing was functional—a diagnosis that should never be lightly made. The paresis and the paræsthesia of the left arm and left leg seemed too definite to allow of any but an organic lesion. The question was, what was the nature of the trouble? The hemiplegic character of the paralytic affection certainly seemed at first sight to indicate that it had its origin in the brain—a syphilitic endarteritis obliterans followed by thrombosis, for example. There was, however, neither history nor other evidence of syphilis. The actual diagnosis, however, was obscure, and it was decided to administer iodide of potassium.

Ten-grain doses of this drug were given three times a day, without mercury, and the patient afforded a good instance of intolerance of the drug by idiosyncrasy. He began taking the iodide on November 20. After the first dose he felt an uneasiness in his head, and after the third dose he began to feel as if he were tipsy. A profuse watery discharge began to flow from his nose, the eyelids became œdematous, with a slight blush of redness; at the same time there was a flow of saliva as copious as if he had been overdosed with mercury. In consequence of these symptoms the iodide was omitted on November 21 and 22, and on the 23rd the iodism had completely subsided. Iodide of potassium was again given in 10-grain doses. Immediately after taking the first dose there was a return of the excessive flow of saliva, which ran from the mouth to the amount of half a pint, measured. The drug was continued notwithstanding this, the ptyalism subsided, and a tolerance was established.

The case reminds one of the aphorism "If iodides produce untoward symptoms, double the dose and the symptoms will subside." Whether or not the iodide was the cause, the patient's condition improved greatly, the severe periodical pain subsided, the man was able to move himself about much better, and he began to regain the power and sensibility of his left arm and leg.

He continued to improve up to December 11, taking the iodide together with 5-grain doses of citrate of iron all the time. He then left the hospital, stating that he could walk better than at any time since his acident. The very day after he left the hospital, however, the pain returned, the patient became gradually worse, and on January 1 he was readmitted. The pain was now continuous; there was tenderness over the lower dorsal and lumbar vertebræ, the pain was much increased by turning in bed or by stooping forward when he sat up. He moved with extreme caution; a sudden jar to his spine was exquisitely painful. It was now clear that he had either tuberculous caries, an aneurysm, or a new growth involving his spine. The weakness of the left arm and left leg had returned, with paræsthesia as before; the limbs of the right side were still natural. Since then the spinal trouble has increased, and it has become clear that the trouble is, and probably had been all the time, very widespread tuberculosis of the vertebræ. He has now developed complete paraplegia of the legs, with anæsthesia, and loss of control over his sphincters.

Looking back at the earlier period of the disease, it is now evident that the tuberculous trouble in his spine must have either extended along on the inside of the vertebral canal, on its left side, from his lower cervical to his lumbar vertebræ continuously, or else there must have been two separate foci of spinal caries, one in the cervico-dorsal region, causing paresis of the arm, the other in the dorso-lumbar region, causing paresis of the leg. Two foci of caries in the spine are not uncommon; but in this particular patient the left arm and left leg always improved or got worse together. It seems more likely, therefore, that there was a long stretch of caseous tubercular material extending from lower cervical to upper lumbar region, and throughout its course bulging from the left side of the canal more than from the right and compressing the left side of the cord.

Be this as it may, the lesion is clearly spinal, the case is one of spinal hemiplegia, and, as in the two former cases we have given, the anæsthesia is on the *same* side as the paralysis.

